# Thromboelastography results on citrated whole blood from clinically healthy cats depend on modes of activation

**DOI:** 10.1186/1751-0147-52-38

**Published:** 2010-06-08

**Authors:** Clara B Marschner, Charlotte R Bjørnvad, Annemarie T Kristensen, Bo Wiinberg

**Affiliations:** 1 Department of Small Animal Clinical Sciences, Faculty of Life Sciences, University of Copenhagen, Denmark, Dyrlægevej 16, DK-1870 Frederiksberg C., Denmark

## Abstract

**Background:**

During the last decade, thromboelastography (TEG) has gained increasing acceptance as a diagnostic test in veterinary medicine for evaluation of haemostasis in dogs, however the use of TEG in cats has to date only been described in one previous study and a few abstracts. The objective of the present study was to evaluate and compare three different TEG assays in healthy cats, in order to establish which assay may be best suited for TEG analyses in cats.

**Methods:**

90 TEG analyses were performed on citrated whole blood samples from 15 clinically healthy cats using assays without activator (native) or with human recombinant tissue factor (TF) or kaolin as activators. Results for reaction time (R), clotting time (K), angle (α), maximum amplitude (MA) and clot lysis (LY30; LY60) were recorded.

**Results:**

Coefficients of variation (CVs) were highest in the native assay and comparable in TF and kaolin activated assays. Significant differences were observed between native and kaolin assays for all measured parameters, between kaolin and TF for all measured parameters except LY60 and between native and TF assays for R and K.

**Conclusion:**

The results indicate that TEG is a reproducible method for evaluation of haemostasis in clinically healthy cats. However, the three assays cannot be used interchangeably and the kaolin- and TF activated assays have the lowest analytical variation indicating that using an activator may be superior for performing TEG in cats.

## Background

Monitoring of coagulation has traditionally been based on prothrombin time (PT) and activated partial thromboplastin time (aPTT). These tests can serve as global screening methods for certain coagulation factors, diagnostically localizing a defective function in either the intrinsic or extrinsic coagulation pathways. However, they provide only limited information on the overall haemostatic capability of the patient [1]. In addition, parameters such as fibrinogen degradation products (FDPs) and D-dimers have been used to evaluate the extent of fibrinolysis with measurement of D-dimers as the only diagnostic test for detection of breakdown of cross linked insoluble fibrin. Despite the ability to detect fibrin/fibrinogen degradation products, these tests have not been recommended as the sole tests to evaluate the role of fibrinolysis in overall haemostasis [2]. Finally, the impact of different cell types on haemostasis is completely lacking in plasma-based tests.

During the last decade, a new model of haemostasis, the cell-based model, has gained increased acceptance. This model is based on the three associated processes: initiation, amplification and propagation followed by fibrinolysis. Initiation takes place on tissue factor (TF)-bearing cells, whereas amplification and propagation occurs on the surface of platelets with thrombin production and fibrin polymerization as the final result [3]. Development of this model has led to a better understanding of physiological and pathological mechanisms influencing the haemostatic balance.

Thromboelastography (TEG) is a point of care technique performed on whole blood, which measures the viscoelastic properties during clot formation and breakdown in a low shear environment [4]. The technique was developed in 1948 and has gained wide use in human medicine over the last decade [5-7]. In veterinary medicine, TEG has been subject to increasing interest in the last few years [8]. The technique has been validated for haemostasis monitoring in dogs, followed by studies proving the clinical relevance of TEG in this species [9-13]. It is expected that the same can be achieved in cats.

A few studies published only as abstracts have suggested that TEG can serve as a diagnostic tool in cats [14-16]. However, to the best of our knowledge, no full studies validating TEG in this species have been published and haemostasis monitoring in cats is therefore currently almost exclusively limited to coagulation tests, which reflect the functionality of the plasma components of the blood such as PT and aPTT. Consequently, evaluation of haemostasis in diseases like feline cardiomyopathy, a disease known to predispose to hypercoagulability and thromboembolism, is difficult due to the lack of diagnostic tests that can reliably identify hypercoagulability and global haemostatic imbalances [17].

The overall purpose of this study was to evaluate analytical performance of three TEG assays: native (without activator added), human recombinant tissue factor (TF) activated and kaolin activated respectively differing from each other only by mode of initiation of coagulation.

## Materials and methods

### Recruitment and sampling

The study was conducted between March 30^th ^and April 30^th ^2009 at the Small Animal Hospital, Department of Small Animal Clinical Sciences, Faculty of Life Sciences (KU-LIFE), University of Copenhagen, and all haematological analyses were performed at The Clinical Pathology Laboratory at the department. The study was approved by the Small Animal Ethics and Administrative Committee. Blood samples were collected from 22 clinically healthy cats belonging either to students or staff or to owners who had previously shown interest in letting their cat participate in clinical studies at the department. Owners were informed about the study by an information letter and were asked to sign a statement confirming their agreement of participation. The health status of the cats was determined by clinical examination as well as unremarkable results on a Complete Blood Count (CBC) (Advia 120, Siemens), a biochemistry panel (Advia 1800, Siemens) and a coagulation profile (ACL TOP 500, Instrumentation Laboratories). All results were assessed by a clinical pathologist.

Blood samples were collected by jugular venipuncture using a 21 Gauge butterfly needle. Blood was collected into one 3 mL serum, two 3 mL trisodium citrate (3.2%, 0.109 M) and one 2 mL EDTA plastic tubes (Vacuette, Greiner Bio-One International AG) in that order. Immediately after collection, the citrated tubes were inverted carefully five times to ensure adequate mixing of citrate and blood. Citrated tubes for TEG analyses were left in a standing position for 30 min at room temperature (20-25°C) before TEG analyses. Standard coagulation profile was performed on citrated plasma after centrifugation at 4400 *g *for 3 min and consisted of an aPTT (aPTT SynthAFax, Instrumentation Laboratories), PT (PT-Fibrinogen, Instrumentation Laboratories), fibrinogen (PT-Fibrinogen, Instrumentation Laboratories) and D-Dimers (D-Dimer Single Tests, NycoCard READER II, Medinor A/S).

### TEG analysis

Analyses were performed using a computerized thromboelastograph (TEG^® ^5000 Haemostasis Analyzer, Haemonetics Corporation). Data was directly transferred to a computer and results were displayed graphically and recorded in a database.

Duplicate whole blood samples were analyzed in parallel using TF, kaolin and native assays respectively for each cat giving a total of six TEG analyses per cat. The analyses were performed in the mentioned order using pre heated TEG cups (37°C) with addition of 20 μL 0.2 M CaCl_2 _prior to addition of citrated whole blood (WB). TF was produced by dilution of human recombinant tissue factor (Innovin, Siemens AG) in a 4-(2-hydroxyethyl)-1-piperazine ethanesulfonic acid (HEPES) buffer with bovine serum albumin. 25 μL diluted TF was mixed with 400 μL citrated WB and 340 μL of this mixture were added to the TEG cups yielding a final TF:WB ratio of 1:50,000. Kaolin activated WB was prepared by adding 1 mL citrated WB to kaolin primed tubes (Haemonetics Corporation) and 340 μL of this mixture was added to the TEG cups. For native analysis 340 μL citrated WB was added directly to the TEG cups.

Immediately after addition of WB, the cup was raised to the pin and measurement was initiated. Six TEG parameters were investigated: R, K, α, MA, LY30 and LY60. R is the time span from initiation until the first fibrin polymers are produced. The cut-off value is when the amplitude reaches 2 mm. K is the time until an amplitude of 20 mm is reached representing the speed of clot formation. α is the angle representing the acceleration and thus kinetics of the fibrin production and cross-linking. MA is the maximum amplitude, reflecting the maximal clot strength [6,18]. LY30 and LY60 are the percent clot lysis detected at 30 and 60 min after MA is reached respectively [18].

The first four parameters reflect different incidents during clotting: R time is dependent on the action of coagulation factors; K time is dependent on the action of coagulation factors, fibrinogen and platelets; the α-angle is dependent on the action of fibrinogen, the platelet function and the platelet membrane composition and MA is dependent on the platelet/fibrin interactions [6,8,18]. LY30 and LY60 percentages depend on the fibrinolytic activity [18].

### Statistical analysis

Statistical analyses were performed using GraphPad Prism 5 (GraphPad Software Inc.).

Coefficients of Variation (CVs) for the four parameters were determined using the arithmetic mean and variance estimate, the latter based on the difference between the duplicate samples.

Distribution of the data was determined using D'Agostino-Paerson normality test. Repeated measures ANOVA followed by a Tukey's Multiple Comparison Test was used to analyze normally distributed data and a Freidman's test followed by a Wilcoxon signed rank test was used to analyze non-parametric data with the null-hypothesis that there was no difference between any of the three assays with regard to the six parameters. Statistical significance level was set at 0.05.

## Results

Of the 22 cats, 7 were excluded due to: missing data (n = 3), ulcerated cutaneous tumour (n = 1), abnormal biochemical results (n = 2) and inadequate blood flow for TEG analysis during blood sample collection (n = 1). CBC, biochemistry and coagulation profiles for the 15 remaining cats were unremarkable. Results for coagulation profiles are shown in Table 1.

**Table 1:** Coagulation profiles in 15 clinically healthy cats

	Fibrinogen (g/L)	PT (sec)	APTT (sec)*	D-dimer (mg/L)
Mean	1.90	10.29	20.24	0.19
Range	1.33-2.62	9.4-11.5	12-33.8	0.1-0.6

The profile includes fibrinogen and D-dimer concentrations. Prothombin time (PT) and activated partial thromboplastin time (APTT) are given in seconds.

*Number of cats = 14.

Of the 15 included cats, 10 were castrated males, 4 were spayed females and 1 was an intact female and consisted of the breeds Domestic Short Hair (n = 9), Birman (n = 2), British Short Hair (n = 1), Maine Coon (n = 1), Siamese (n = 1), and Rag Doll (n = 1). The mean age of the study population was 3.3 y (0.11-7.9 y).

The normality test revealed that results obtained for the parameters K and LY30 were not normally distributed. The arithmetic mean, range and the CV for the six parameters are shown in Table 2. There were statistically significant differences between the three assays for R (*P *< 0.0001), K (*P *= 0.0027), α (*P *= 0.0003), MA (*P *< 0.0001), LY30 (*P *= 0.0077), and LY60 (*P *= 0.001). Tukey's Multiple Comparison Test of R, α, MA, and LY60 showed significant differences (*P *< 0.05) between native and kaolin for all four parameters, between native and TF for R but not for α, MA or LY60 and between TF and kaolin for R, α, and MA, but not for LY60. Wilcoxon signed rank test showed that there was significant differences for K between native and TF (*P *= 0.0052), native and kaolin (*P *= 0.002) and between TF and kaolin (*P *= 0.0041) and for LY30 between native and kaolin (*P *= 0.0076) and TF and kaolin (*P *= 0.0049) but not between native and TF.

**Table 2:** Thromboelastograph measurements in 15 clinically healthy cats

Group		R minutes	K^†^ minutes	α degrees	MA mm	LY30^†^ %*	LY60 %*
Native	Mean	10.06	3.55	49.23	50.83	0.18	2.97
	Range	3.3-19.8	1.9-6.6	32.5-64.6	38.3-60.7	0.0-4.0	0.0-8.9
	CV (%)	9.96	15.04	7.76	4.09	193.93	100.31
		
TF	Mean	7.46	2.85	53.20	52.41	0.30	4.25
	Range	3.2-12.5	1.9-5.8	34.1-64.3	40.3-62.8	0.0-5.6	0.1-10.5
	CV (%)	8.29	11.34	7.08	2.86	25.12	14.15
		
Kaolin	Mean	5.57	2.00	61.83	56.61	1.05	5.41
	Range	2.4-9.5	1.2-3.9	45.5-73.5	46.8-66.1	0.0-9.0	1.1-13.1
	CV (%)	6.42	14.80	3.65	2.57	17.54	12.10

Measurements were done without activator (native) as well as with tissue factor (TF) or kaolin as activators.

R = reaction time; K = clotting time; α = angle; MA = maximum amplitude; LY30 = clot lysis 30 min after MA is reached; LY60 = clot lysis 60 min after MA is reached.

^†^Median.

*Mean, range and CV% calculated using the results obtained in 14 cats. TEG analyses on feline citrated whole blood in our department prior to this study have occasionally revealed a distinct TEG trace pattern showing increased LY values, a finding that has been encountered previously [16]. In this study the encountered pattern in question only appeared once leading to exclusion of the LY data of one cat.

## Discussion

The present study demonstrates that TEG is a reproducible method for evaluation of haemostasis in clinically healthy cats, when any of the three assays native, TF or kaolin is used. Statistical analyses showed that there are significant differences between all six parameters when comparing the native and kaolin assays. Likewise, all parameters are significantly different when comparing TF and kaolin assays with the exception of LY60, whereas the difference between the TF activated and native assays is related solely to the initiation phase of coagulation (R and K). The study suggests that the results obtained with the three assays cannot be used interchangeably. Of the three assays, native has the highest CV in all six parameters, whereas kaolin has the lowest CV in all six parameters. The finding of a larger analytical variation in the results using the native assay compared to TF based or kaolin based assays was expected especially regarding the R and K parameters since initiation of coagulation in the native assay occurs spontaneously and not as a result of activation by a standardized amount of either TF or kaolin. The finding of a high variation using the native assay correlates well with the results obtained in a previous study in cats [19]. In addition, the CVs for R, K, α, and MA for the TF based and kaolin based assays calculated in our study are well in accordance with CVs reported when using TF activated TEG in dogs as well as using kaolin and TF activated TEG in humans [9,20].

It was expected that the kaolin based assay would differ significantly from the TF based assay. Significant differences between kaolin and TF activated TEG have been reported for K, α and MA in a previous study [15]. The results obtained in the present study show that kaolin based assays differ from TF based assays by significantly shorter R and K values as well as significantly higher α and MA values suggesting that kaolin is a more potent activator than diluted human recombinant TF in cats. Mechanisms by kaolin and TF activation on the coagulation in cats remain to be investigated. Since TF is recognized as the initiator of coagulation *in vivo *according to the cell based model of haemostasis, TF activated TEG analyses may provide a better *in vitro *estimate of *in vivo *coagulation. On the other hand the kaolin based assay is the safest method regarding risks of pre-analytical errors, since ready-to-use kaolin tubes are provided by the manufacturer and thus easily accessible, whereas human recombinant TF must be prepared from a concentrated stock on a daily basis, leading to a larger risk of inter-assay variation when using this assay.

The results for K, α, and MA in the native assay are comparable to findings obtained in one other study using native TEG in cats [16]. However, only MA results are similar when compared to a second study using the native TEG assay [19]. Since both these studies are published in abstract form only, a thorough comparison of the methods used and thus results obtained is difficult.

## Conclusions

The results indicate that TEG is a reproducible method for evaluating haemostasis in clinically healthy cats. The present study shows that TEG analyses using TF- or kaolin based assays are likely to be more reliable than results obtained using the native assay. The results from the present study, with regard to analytical variation of LY values, indicate that the native assay is inferior when compared to the TF based and kaolin based assay. It must be emphasized that comparison of results obtained from the three different TEG assays should be avoided due to the significant differences between the assays documented in the present study. It is therefore recommended that each laboratory use only one assay for routine analyses in order to keep a standardized working procedure. Prospective studies are needed to evaluate the clinical usefulness of the three assays in cats.

## Competing interests

The authors declare that they have no competing interests.

## Authors' contributions

CBM designed the study, carried out the data collection and analysis and drafted the manuscript. CRB and ATK participated in the study design. BW participated in the study design, data analysis and performed the statistical analysis. All authors read and approved the final manuscript.
